# ID 77 - Perfil de Competência do Profissional em Políticas Informadas por Evidências no Brasil

**DOI:** 10.5327/2237-9622.2025.v34s1.77

**Published:** 2025-11-25

**Authors:** Jorge Otávio Maia Barreto, Davi Mamblona Marques Romão, Cecilia Setti, Maria Lúcia Teixeira Machado, Rachel Riera, Romeu Gomes, Daienne Amaral Machado, João Abreu, Keitty Regina Cordeiro de Andrade, Laura dos Santos Boeira, Letícia Pozza, Nathan Mendes Souza, Patrícia Logullo, Roberta Borges Silva, Sandra Maria do Valle Leone de Oliveira, Sara Emanuela de Carvalho Mota, Tamille Sales Dias, Tereza Setsuko Toma, Silvio Fernandes da Silva

**Keywords:** política informada por evidências, tomada de decisões, tradução do conhecimento, perfil de competência, conhecimentos, habilidades e atitudes

## Abstract

**Introdução:**

A formulação de políticas informadas por evidências (PIE) requer um conjunto de conhecimentos, habilidades e atitudes individuais e organizacionais que devem ser articulados com fatores e necessidades de contexto. Nesse sentido, o desenvolvimento de um perfil de competência PIE é importante para dar suporte ao diagnóstico, ao planejamento e à implementação do PIE. O objetivo deste resumo é apresentar o processo e os resultados do desenvolvimento de um perfil de competência PIE, realizado por um comitê de especialistas e que possa ser aplicado em diferentes contextos do SUS, incluindo usuários de informações relativas à Avaliação de Tecnologias em Saúde (ATS).

**Método:**

Um comitê de especialistas em PIE compartilhou diferentes visões, experiências e opiniões para desenvolver um perfil de competência PIE para o Brasil. Em seis workshops de consenso mediados por facilitadores, o comitê definiu os problemas macro para ações-chave e desempenhos essenciais para o perfil de competência. As etapas de desenvolvimento consistiram em: (1) Constituição do comitê, incluindo pesquisadores, profissionais com experiência prática, gestores e educadores; (2) Desenvolvimento de uma revisão rápida sobre perfis de competência PIE; (3) Acordo sobre compromissos e responsabilidades nos processos; (4) Identificação e definição de problemas macrorrelacionados ao escopo do perfil de competência; e (5) Delineamento de capacidades gerais e específicas a serem incorporadas ao perfil de competência, categorizadas por ações-chave.

**Resultados:**

O desenvolvimento do perfil de competência do PIE foi guiado pelos seguintes problemas macro: (1) falta de processos de tomada de decisão sistemáticos e transparentes na gestão de políticas de saúde; (2) capacidade institucional subdesenvolvida para gestão e tradução do conhecimento; e (3) uso incipiente de evidências científicas na formulação e na implementação de políticas de saúde. Foi desenvolvido um arcabouço geral de ações e desempenhos-chave do Perfil de Competência em PIE para o Brasil, incluindo 42 ações-chave específicas e gerais distribuídas por área de atividade (Gestão em Saúde, Pesquisa Científica, Sociedade Civil, Tradução do Conhecimento e Áreas Transversais).

**Conclusão:**

Muitos estudos abordam elementos teóricos e operacionais de competência relacionados a PIE, mas o processo de contextualização conduzido pelo comitê de especialistas possibilitou ressignificar esses elementos para o contexto nacional brasileiro, uma vez que todo o processo considerou fortemente a experiência e o conhecimento dos participantes do comitê. Essa contextualização do processo também pode ser considerada em perspectiva para confrontar e interpretar os resultados aqui apresentados diante de outras iniciativas de categorização de elementos de competência para diferentes contextos. Este perfil de competência deve ser usado como uma matriz inicial para definir perfis específicos, podendo modular, priorizar e alterar o perfil geral, adicionando ou removendo desempenhos, a fim de torná-lo adequado às necessidades organizacionais concretas. Com base nessas adaptações, é possível desenhar um perfil para cargos e funções específicas, bem como perfis de competência para atividades de treinamento.

